# Effects of hanger reflex on the cervical muscular activation and function: A surface electromyography assessment

**DOI:** 10.3389/fphys.2022.1006179

**Published:** 2022-10-11

**Authors:** Dian Wang, Baoge Liu

**Affiliations:** Department of Orthopaedic Surgery, Beijing Tiantan Hospital, Capital Medical University, Beijing, China

**Keywords:** muscular function, hanger reflex, electrophysiology, surface electromyography, co-contraction ratio, cervical stability, neuromuscular disorder

## Abstract

**Introduction:** Cervical muscular dysfunction is closely associated with disorders and neuromuscular diseases of the cervical spine, and the hanger reflex (HR) has the potential to become a rehabilitation method. The muscular electrophysiology mechanism of HR is unclear. This study aims to identify the impacts of HR on cervical rotators’ myoelectrical activity and function.

**Methods:** We designed a self-control clinical trial, and asymptomatic volunteers were continuously included from 1 September 2021 to 30 April 2022 in our department. Rotation tasks were performed on both sides under each of the situations: no HR, unilateral HR, and bilateral HR. Surface electromyography (SEMG) was used to detect the myoelectrical activity of agonistic splenius capitis (SPL), upper trapezius (UTr), and sternocleidomastoid (SCM). The co-contraction ratio (CCR) during rotation tasks was calculated. Correlation analyses and multiple linear regression were performed.

**Results:** Finally, 90 subjects were enrolled (power >90%). The adjusted EMG value (aEMG) of SPL UTr, SCM, and rotating CCR under the unilateral HR and bilateral HR were higher than no HR; the aEMG of SPL and rotating CCR under the bilateral HR were higher than the unilateral HR. Multiple linear regression showed that HR pattern and age were the independent affecting factors for the aEMG of SPL (*p* < 0.001, *p* < 0.001), UTr (*p* < 0.001, *p* < 0.001), and SCM (*p* < 0.001, *p* < 0.001); BMI was an independent affecting factor for the aEMG of SPL (*p* < 0.001) and SCM (*p* < 0.001); HR pattern was the only affecting factor for CCR (*p* < 0.001).

**Conclusion:** HR can increase the cervical rotators’ myoelectrical activities and rotating CCR, and the effects of bilateral HR are greater than unilateral HR, suggesting that bilateral HR has a greater clinical potential to become a rehabilitation method for treating cervical neuromuscular disorders.

## Introduction

Cervical muscular function is of great significance to the cervical stability, alignment, head posture, and motion (Cheng et al., 2011; Thakar et al., 2014; Ding et al., 2020). Cervical muscular dysfunction can contribute to a series of disorders and neuromuscular diseases, such as neck pain (NP) (Cheng et al., 2011), cervical dystonia, and cervical bending and rotation deformities (Hussein et al., 2017), as well as abnormal postures, such as wryneck posture. Rehabilitation and non-invasive treatments are always needed for treating the cervical disorders related to muscular dysfunction.

The hanger reflex (HR) is an involuntary head rotation that occurs in response to a clothes hanger encircling the head, which compresses the unilateral frontal-temporal area (Asahi et al., 2018). In this situation, the head will rotate towards the compressed side spontaneously, which is termed as the HR (Asahi et al., 2018; Agyei et al., 2019; Asahi et al., 2020). HR is a general phenomenon in populations, and it can be observed in 92.1% of the healthy subjects (Asahi et al., 2015). The mechanism of HR is closely related to the deep sensation-associated cervical muscular activation. It has been reported that HR can correct the abnormal head position in patients with cervical dystonia (Asahi et al., 2018), and HR can be used as a potential rehabilitation method for cervical muscular dysfunction and disorders (Asahi et al., 2018; Asahi et al., 2020).

To our knowledge, the potential mechanism of HR on the cervical rotators is still unclear. Although previous studies have proved the HR phenomenon and estimated the efficacy on several kinds of muscular diseases, the muscular electrophysiology mechanism of HR is still unclear. The superficial and deep cervical rotators synergistically contribute to the posture control and rotation movement (Panjabi et al., 1998; Cheng et al., 2011; Agyei et al., 2019). This study aims to identify the impacts of HR on myoelectrical activity of cervical rotators and neuromuscular function, as well as the affecting factors. We hypothesized that: 1) the subject’s general characteristics may have impacts on the cervical rotators’ myoelectrical activity; 2) the different HR patterns may be an independent affecting factor of the cervical rotators’ electrophysiology function.

## Materials and methods

### Subjects and enrollment

This was a self-control clinical trial, and asymptomatic volunteers were continuously included from 1 September 2021 to 30 April 2022 in our department.

The inclusion criteria were as follows: 1) asymptomatic volunteer; 2) 20–65 years, BMI＜28. The exclusion criteria were as follows: 1) Parkinson’s disease, amyotrophic lateral sclerosis, and the other neuromuscular diseases (Ding et al., 2020); 2) history of spinal deformity, spinal tumor, or compression fracture; 3) surgery history of spine; 4) moderate neck pain with a visual analogue scale (VAS) > 3 (Falla et al., 2007); 5) can not cooperate to the surface electromyography (SEMG) test.

The Institutional Review Board of our hospital has approved human subject protection programs and procedures (Ethic number, KY 2020–073-02). Informed consent was acquired from all participants. The general characteristics included sex, age, height, weight, and BMI.

### Unilateral and bilateral hanger reflex

An ordinary wire clothes hanger was used for the HR, which was flexible and large enough to encircle the subject’s head. The HR of present study consisted of a unilateral HR and a bilateral HR.

**FIGURE 1:**
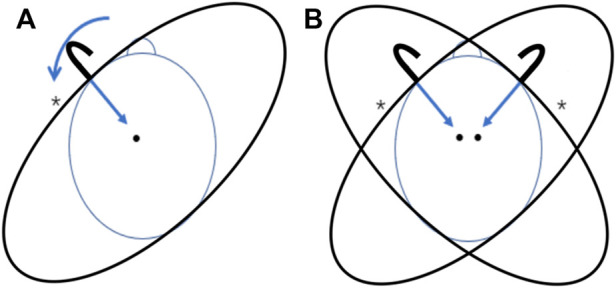
The unilateral and bilateral HR. **(A)** Unilateral HR on the left side, the hanger was placed on the left frontal-temporal area; **(B)** Bilateral HR, two hangers were placed on both sides of the frontal-temporal areas; *, the compressed side of HR.

In the unilateral HR, the hanger was placed on a unilateral frontal-temporal area, and the subject’s head would rotate to the compressed side spontaneously due to the compression (Figure 1A). In the bilateral HR, two hangers were placed on both sides of the frontal-temporal areas (Figure 1B).

### Rotation task

The rotation task comprised 3 phases of movement: the resting phase (phase I), axial rotation to one side (phase II, rotating phase, subject was asked to rotate to the compressed side), and rotation to the neutral position (phase III, returning phase, subject was asked to rotate to the neutral position), and each movement phase lasted 4 s at a constant speed process (Cheng et al., 2008).

**FIGURE 2:**
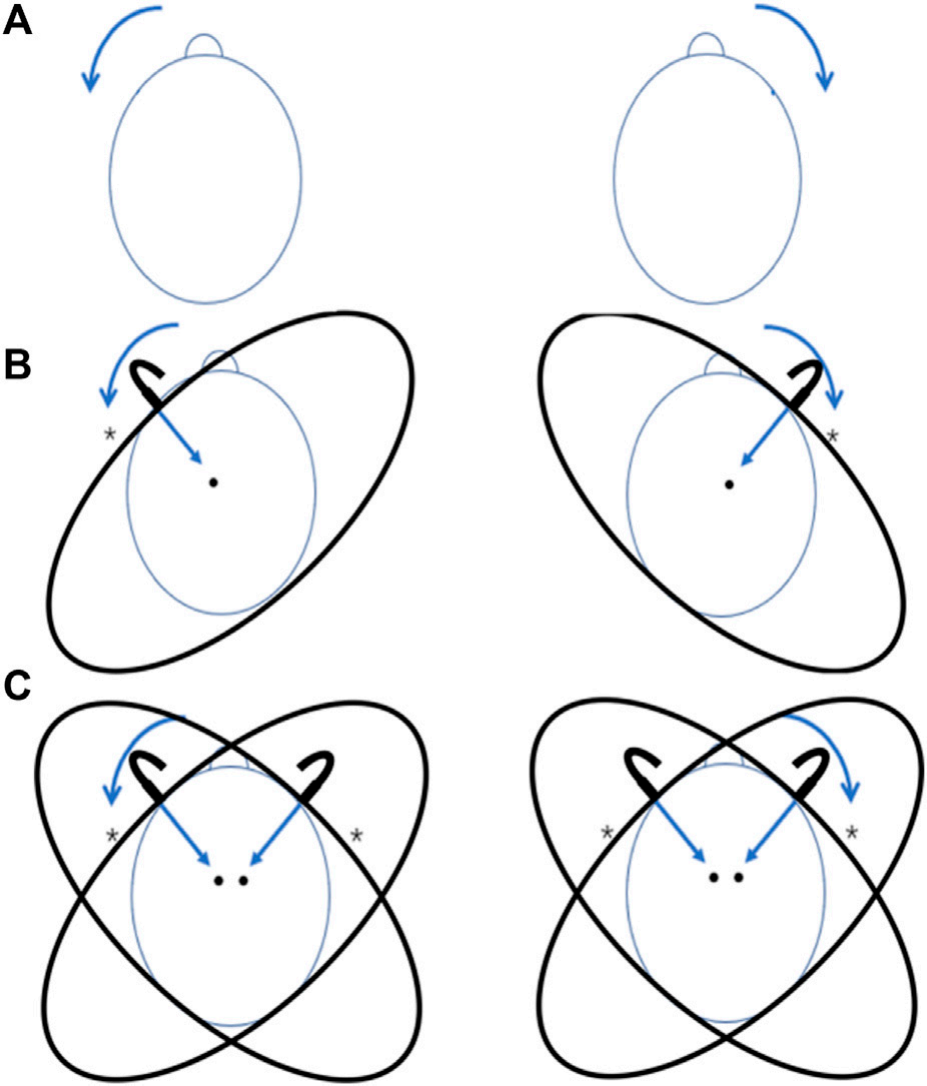
The rotation tasks at on no HR, unilateral and bilateral HR. **(A)** Subjects were asked to start the rotation tasks firstly on the left then the right; **(B)** The compressed sides of unilateral HR were set firstly on the left then the right, and subjects were asked to rotate their heads to the compressed side respectively (first the left then the right); **(C)** In bilateral HR, subjects were asked to start the rotation tasks firstly on the left then the right; *, the compressed side of HR; brow peak, the rotation direction.

Rotation tasks were performed on both sides (first the left then the right) under each of the situations: no HR (Figure 2A), unilateral HR (Figure 2B), and bilateral HR (Figure 2C). The unilateral HR was started on the left side (the compressed side was set on the left side firstly) and then transferred to the right side (Figure 2B). The bilateral HR was also started on the left side, followed by the right side with bilateral compression (Figure 2C). 1 subject provided 2 data (left and right), and both were from the compressed sides (first the left then the right).

### SEMG-based muscle electrical activities and co-contraction ratio

**FIGURE 3:**
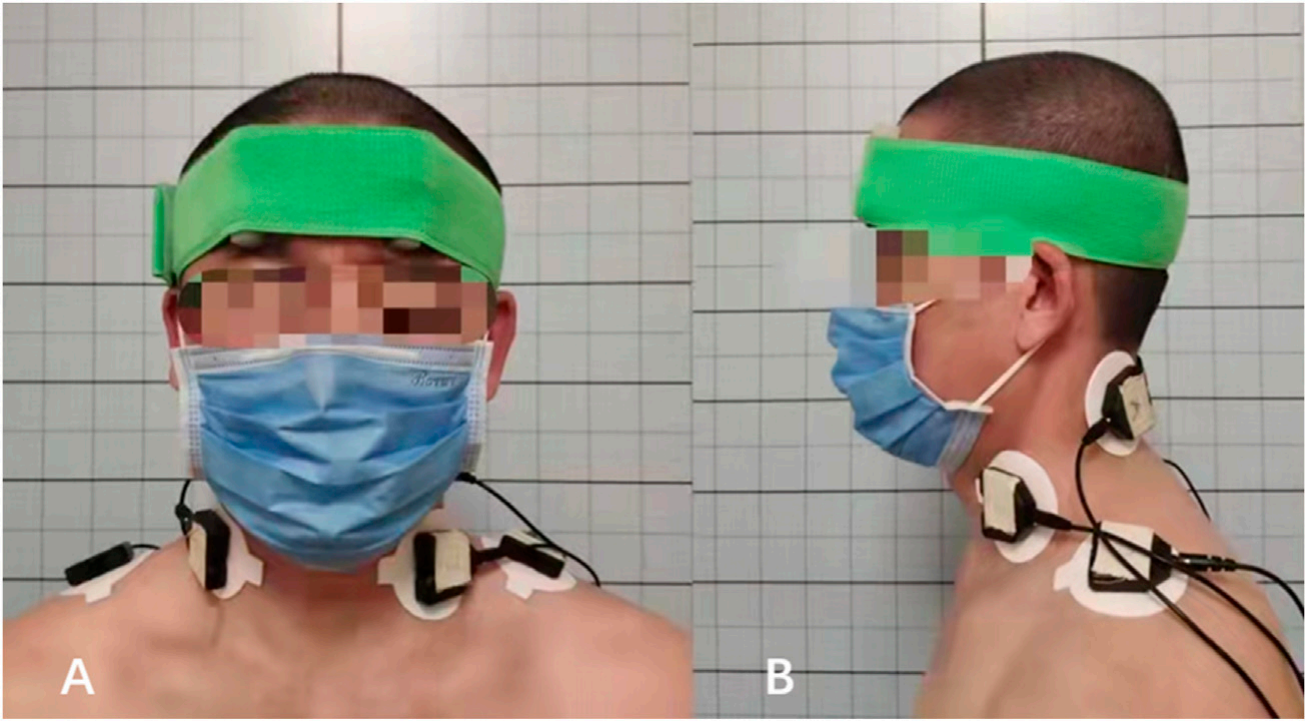
SEMG test of the cervical rotators. **(A)** The anterior view on the surface location of the electrodes, the electrode for SCM was located at the midpoint of the muscle; **(B)** The lateral view on the surface location of the electrodes, the electrode for SPL was located 2 cm from the spinous process at the C4 level, and electrode for UTR was located at the midpoint of a line between the C7 spinous process and the middle of the acromion.

SEMG is an objective and non-invasive tool for detecting myoelectrical activities and assessing muscular function (Colloca and Hinrichs, 2005; Marshall and Murphy, 2006). The myoelectrical activity of splenius capitis (SPL), upper trapezius (UTr), and sternocleidomastoid (SCM) were recorded with a SEMG device (FlexComp Infiniti System, T7550, Thought Technology Ltd, Canada) during the rotation tasks. The electrodes (Ag–AgCl electrode, diameter 2 cm, CH55RD, Cathay manufacturing group, Shanghai, China) for SPL were located 2 cm from the spinous process at the C4 level (Joines et al., 2006; Ding et al., 2020), and the electrodes for UTR were located at the midpoint of a line between the C7 spinous process and the middle of the acromion (Ding et al., 2020), the electrodes for SCM were located at the lower one-third of the mastoid process and sternal notch (Falla et al., 2002) (Figure 3).

All subjects were asked to perform the rotation task (rotated to left/right sides) for 6 times: with no HR, with unilateral HR (compressed side: left/right), and with bilateral HR. There was a 2-min break between each task to minimize the effects of fatigue (Cheng et al., 2008). To familiarize the subjects with the movement phases and speeds, sufficient practice was needed. The SEMG signal was 1000 Hz and was band-pass filtered between 20 and 450 Hz. The electromyogram (EMG) of SPL, UTr, and SCM (agonists) on the ipsilateral side of the rotating orientation were recorded during the tasks, and the root mean square (RMS) values of EMG were calculated automatically by the system. Finally, the adjusted EMG value (aEMG) of SPL, UTr, and SCM under no HR, unilateral HR, and bilateral HR were calculated. The aEMG = the maximal activity during the rotating phase - mean activity during the returning phase.

The sEMG-based CCR is an objective and quantified method for assessing the co-contraction pattern of the muscular system of cervical spine (Cheng et al., 2008; Chih-hsiu et al., 2014). CCR is the reference for the coordination of agonists and antagonists in a balanced muscular system. The maximal voluntary isometric contraction (MVIC) of SPL, UTr, and SCM was recorded for 3-s. The normalized average integration EMG (NAIEMG) during the rotating phase was calculated, using the central 1-s MVIC as reference (Chih-hsiu et al., 2014). The rotating CCR under no HR, unilateral HR, and bilateral HR were calculated. CCR = NAIEMG of the agonists (ipsilateral SPL+UTr+SC)/ NAIEMG of total muscle (ipsilateral and contralateral SPL+UTr+SC).

### Statistical analysis

The sample size was calculated with a formula: n=(μα+ μβ)^2^ σ^2^/ δ^2^ (for paired sample *t*-test), α was set as 0.05, and β was set as 0.01. According to the μ Table, values of μα and μβ were 1.96 and 1.2816 respectively. Values of σ (25.83) and δ (10.77) came from the preliminary experiment (n = 26, aEMG of SPL without HR and with unilateral HR). The calculated sample size was 60.4, which was further amplified by 20%–72.5.

All of the statistical analyses were performed using IBM SPSS 20.0.0 (SPSS Inc. 2009; Chicago, IL, United States). Measurement data were checked for normal distribution by Kolmogorov-Smirnov test. Comparisons between groups (no HR, unilateral HR, bilateral HR) were processed by paired sample *t*-tests and Levene variance homogeneity tests. Correlations between parameters were performed by Spearman analysis. Multiple linear regression analysis was performed to determine the independent factors of aEMG and CCR. ROC (receiver operating characteristic) curves were performed in order to determine the cut-off values of indicators. The level of significance was set at 0.05.

## Results

### General characteristics

The calculated sample size was further amplified by 20%–72.5. Finally, we enrolled 90 subjects (power >90%), and all of those subjects had accomplished the whole study. The basic characteristics of the subjects were listed below, and all of them obeyed the normal distribution (Table 1).

**TABLE 1:** The basic characteristics of the subjects.

Basic characteristic	Asymptomatic subjects
Number (case)	90
Sex (male/female)	36/54
Age (years)	40.35±16.45 (range: 20–65)
Height (m)	1.66±0.11 (range: 1.44–1.91)
Weight (kg)	64.46±6.91 (range: 50.19–84.63)
BMI (kg/ cm^2^)	23.38±1.6 (range: 19.05–26.89)

### The aEMG of cervical rotators

**FIGURE 4:**
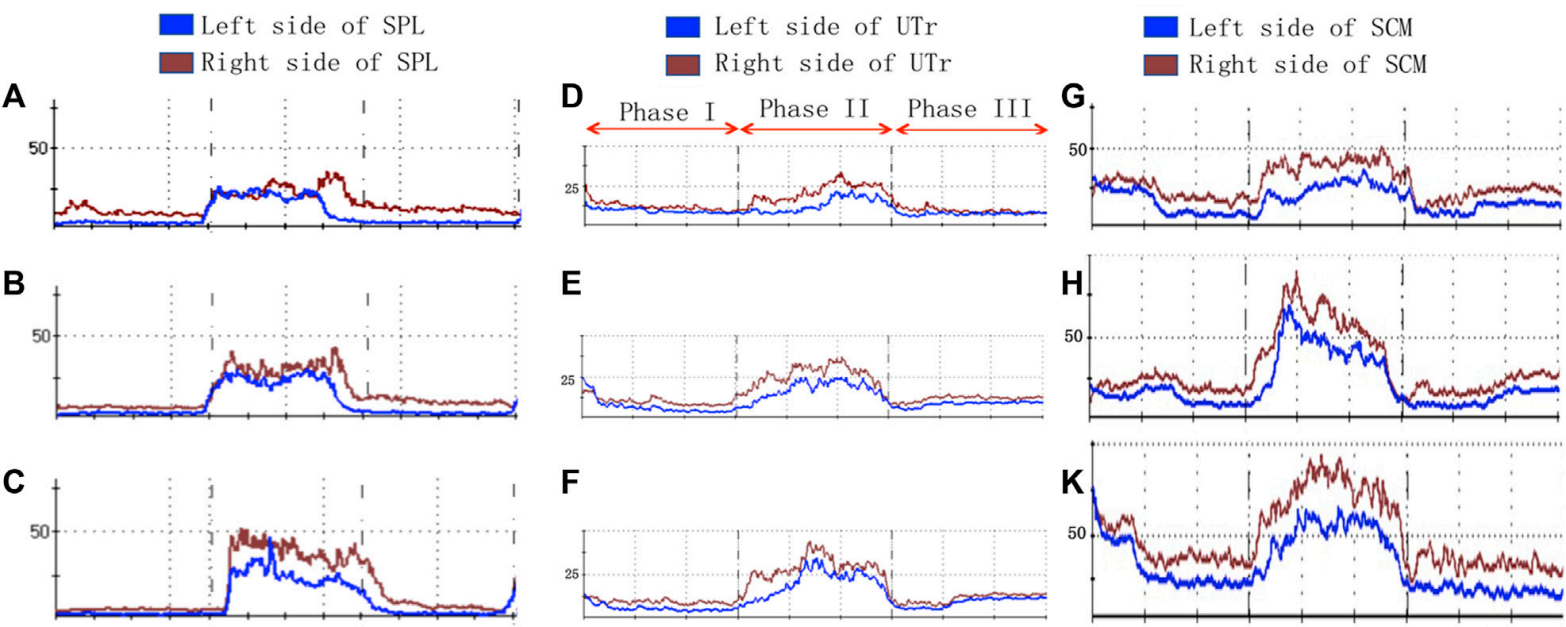
SEMG-based myoelectrical activities of the cervical rotators under the different HR patterns. **(A–C)**, no HR; **(D–E)**, unilateral HR; **(G–K)**, bilateral HR; Phase I, resting phase; Phase II, rotating phase; Phase III, returning phase.

EMG test showed that the RMS of SPL, UTr, and SCM were increased notably under the unilateral HR and bilateral HR, comparing to the no HR (Figure 4). The aEMG of those rotating agonists were calculated by using the maximal EMG during the rotating phase (Figure 4D) minus the mean EMG during the returning phase (Figure 4D). The aEMG of SPL, UTr, SCM, as well as the CCR under the unilateral HR were higher than the no HR situation (Table 2). The aEMG of SPL and CCR under the bilateral HR were higher than the unilateral HR (Table 2).

**TABLE 2:** The aEMG and CCR under no HR, unilateral HR and bilateral HR.

Parameters	No HR (A)	Unilateral HR (B)	Bilateral HR (C)	A vs. B	B vs. C
aEMG of SPL	29.11±13.72	33.48±18.14	40.58±22.91	*t* = -2.595	*t* = -3.278
				*p* = 0.010*	*p* = 0.001**
aEMG of UTr	12.69±6.90	14.74±8.75	16.35±9.91	*t* = -2.491	*t* = -1.639
				*p* = 0.013*	*p* = 0.102
aEMG of SCM	41.97±20.12	54.57±36.18	58.84±35.28	*t* = -4.102	*t* = -1.140
				*p* < 0.001**	*p* = 0.225
CCR	0.50 ±0.01	0.51±0.02	0.53±0.02	*t* = -7.271	*t* = -7.125
				*p* < 0.001**	*p* < 0.001**

aEMG, adjust electromyogram; CCR, co-contraction ratio; SPL, splenius capitis; UTr, upper trapezius; SCM, sternocleidomastoid; *, *p* < 0.05 **, *p* < 0.01.

### Correlation analyses

Correlation analyses were performed between the HR pattern (no HR = 0, unilateral HR = 1, bilateral HR = 2), aEMG of the agonists rotators, CCR, and the general characteristics (Table 3). The HR pattern had mild correlations with the aEMG of SPL, UTr, and SCM, and it had a moderate correlation with the CCR. Mild correlations existed commonly between the general characteristics including sex, age, height, weight, BMI, and the aEMG of rotating agonists (Table 3).

**TABLE 3:** Correlation analysis of the aEMG, CCR and general characteristics.

Group		Sex	Age	Height	Weight	BMI	SPL aEMS	UTr aEMS	SCM aEMS	CCR
Group	1	0	0	0	0	0	0.222**p < 0.001	0.137**p = 0.001	0.201**p < 0.001	0.582**p < 0.001
Sex		1	−0.009 *p* = 0.834	0.468**p < 0.001	0.360**p < 0.001	−0.362**p < 0.001	0.092*p = 0.031	0.081 *p* = 0.060	0.139**p = 0.001	−0.002 *p* = 0.958
Age			1	0.006 *p* = 0.889	0.328**p < 0.001	0.540**p < 0.001	0.045 *p* = 0.294	0.370**p < 0.001	0.151**p < 0.001	0
Height				1	0.865**p < 0.001	−0.575**p < 0.001	0.180**p < 0.001	0.140**p = 0.001	0.229**p < 0.001	−0.014 *p* = 0.741
Weight					1	−0.122**p = 0.004	0.155**p < 0.001	0.265**p < 0.001	0.258**p < 0.001	−0.030 *p* = 0.477
BMI						1	−0.102*p = 0.017	0.168**p < 0.001	−0.196**p < 0.001	−0.013 *p* = 0.754
SPL aEMS							1	0.079 *p* = 0.066	0.146**p = 0.001	0.078 *p* = 0.067
UTr aEMS								1	0.151**p < 0.001	0.083 *p* = 0.052
SCM aEMS									1	0.169**p < 0.001
CCR										1

aEMG, adjust electromyogram; CCR, co-contraction ratio; SPL, splenius capitis; UTr, upper trapezius; SCM, sternocleidomastoid; HR pattern, no HR = 0, unilateral HR = 1, bilateral HR = 2; Sex, male = 1, female = 0; *, *p* < 0.05 **, *p* < 0.01.

### Multiple linear regression analysis

The multiple linear regression of aEMG showed that the HR pattern and age were the independent affecting factors for the aEMG of SPL, UTr, and SCM, and the subject’s BMI was also an independent affecting factor for the aEMG of SPL and SCM (Table 4). The multiple linear regression of CCR showed that the HR pattern was the only affecting factor for the rotating CCR of the cervical spine (Table 5).

**TABLE 4:** Multiple linear regression analysis for aEMG of the cervical rotators.

Factors	SPL aEMG (R = 0.345)				UTr aEMG (R = 0.428)				SCM aEMG (R = 0.416)			
	B	SEM	*P*	VIF	B	SEM	*p*	VIF	B	SEM	*p*	VIF
HR pattern	5.738	0.948	<0.001**	1.000	1.831	0.415	<0.001**	1.000	8.431	1.538	<0.001**	1.000
Sex	0.535	1.753	0.761	1.260	1.347	0.767	0.080	1.260	1.273	2.845	0.655	1.260
Age	0.249	0.063	<0.001**	1.782	0.227	0.027	<0.001**	1.782	0.672	0.102	<0.001**	1.782
BMI	−3.604	0.694	<0.001**	2.057	−0.485	0.303	0.110	2.057	−8.727	1.126	<0.001**	2.057

aEMG, adjust electromyogram; SPL, splenius capitis; UTr, upper trapezius; SCM, sternocleidomastoid; SEM, standard error of mean; VIF, variance inflation factor; **, *p* < 0.01.

**TABLE 5:** Multiple linear regression analysis for the cervical rotating CCR.

Factors	B	CCR (R = 0.345)		
		SEM	*P*	VIF
HR pattern	0.015	0.001	<0.001**	1.160
Sex	8.18*10^−5^	0.002	0.962	1.268
Age	1.22*10^−5^	0.000	0.859	2.201
BMI	0.000	0.001	0.632	2.399
SPL aEMG	−4.78*10^−5^	0.000	0.261	1.135
UTr aEMG	−2.00*10^−5^	0.000	0.837	1.225
SCM aEMG	2.15*10^−5^	0.000	0.412	1.210

CCR, co-contraction ratio; aEMG, adjust electromyogram; SPL, splenius capitis; UTr, upper trapezius; SCM, sternocleidomastoid; SEM, standard error of mean; VIF, variance inflation factor; **, *p* < 0.01.

### ROC curve analysis and the cut-off values

**FIGURE 5:**
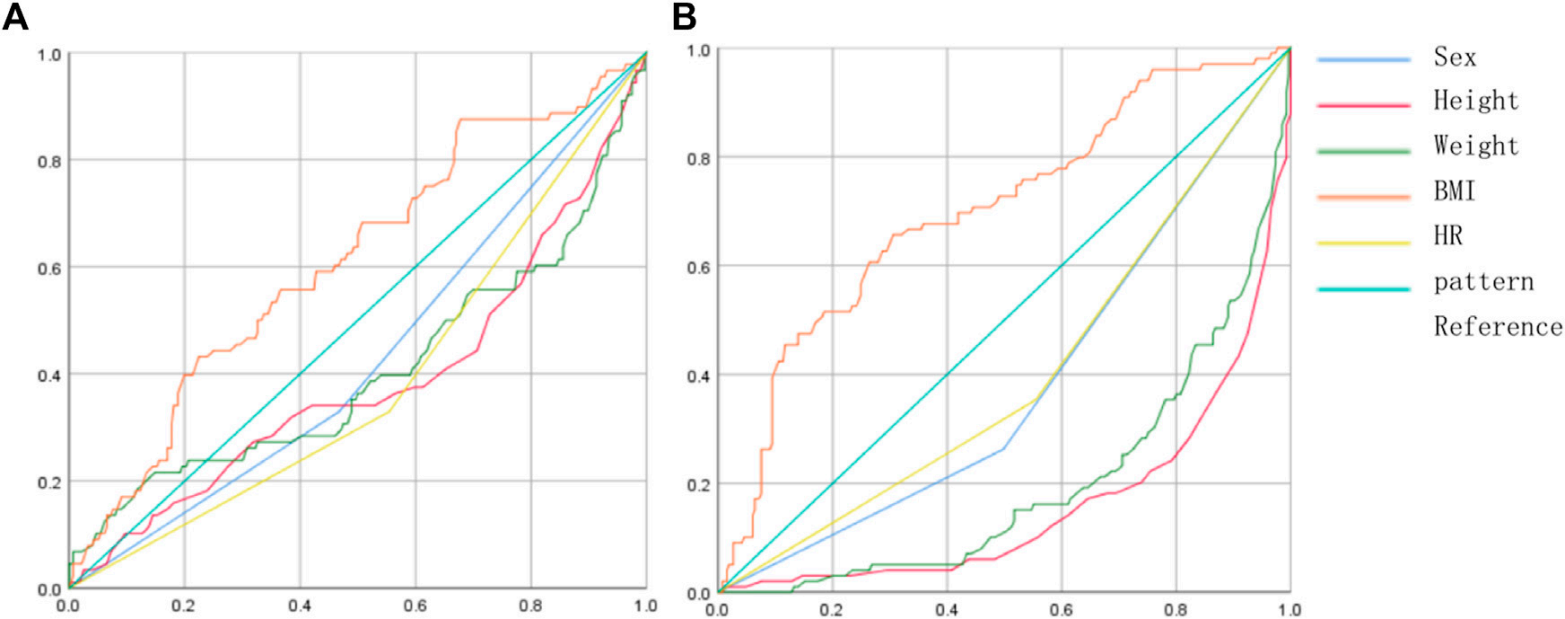
ROC curves of BMI and aEMG of the SPL and SCM. **(A)** ROC curves of the factors and SPL aEMG, the orange color represented the BMI; **(B)** ROC curves of the factors and SCM aEMG, the orange color represented the BMI.

The ROC analysis showed that the cut-off value of BMI for the aEMG of SPL was 24.37 (*p* < 0.05) (Figure 5A), and the cut-off value of BMI for the aEMG of SCM was 23.99 (*p* < 0.05) (Figure 5B).

## Discussion

SPL, UTr, and SCM are the main rotators of the cervical spine, which play a critical role in maintaining normal posture, alignment, and spinal stability (Vasavada et al., 1998). SPL is a deep layer rotator, and UTr and SCM are superficial rotators. The superficial and deep rotators synergistically contribute to the posture control and rotating motion (Panjabi et al., 1998; Cheng et al., 2011; Agyei et al., 2019). HR is a general phenomenon in populations. It first came to light publicly in 1995 in Japan when a television program reported that a man who wore a wire hanger around his head subsequently rotated his head (Agyei et al., 2019), and this phenomenon was formally termed as the “HR” by Kajimoto et al. at the University of Electro-Communications in 2008 (Matsue et al., 2008). An epidemiological investigation by Asahi T et al. found that HR is a general phenomenon in populations (120 healthy Japanese adults, 60 men and 60 women, aged 19–65 years), and it can be observed in 92.1% of the healthy subjects (Asahi et al., 2015). Till now, the potential mechanism of HR remains unknown. Previous study explained the HR phenomenon as the unilateral muscular activation of cervical rotators caused by imbalance stress (deep sensation) in the frontal-temporal area, and the subject feels an urge to rotate the head to the compressed side, in order to maintain an original position and a balanced posture (Asahi et al., 2020). However, none of them have explored the effects of HR on the muscular system, such as SEMG or kinematic study.

In this study, we observed the immediate electromyography activations of the cervical rotators in two patterns of HR. To our knowledge, it was the first study focusing on the electromyography mechanism of HR and the cervical rotator function. Our results found that the aEMG of SPL, UTr, and SCM were significantly increased, both under the unilateral and bilateral HR, however, the aEMG of SPL was further increased under the bilateral HR, compared with the unilateral HR. It indicated that HR can activate the cervical rotators by increasing their myoelectrical activities during the active rotation, and the effects of bilateral HR were stronger than unilateral HR. It has been reported that HR-associated muscular activation can be used as a non-invasive treatment for some neuromuscular disorders with imbalanced muscular strength and posture, such as cervical dystonia, NP, and adhesive capsulitis (Agyei et al., 2019). It has been reported that unilateral HR can immediately correct the abnormal posture and improve the symptoms in cervical dystonia patients (Asahi et al., 2010). A pilot clinical study also found that an HR-simulated device (30 min/day, 3 months) can significantly improve the symptoms and subjective severity scale in 19 patients with cervical dystonia, both at the baseline and after the 3-month trial, suggesting that HR can improve abnormal head rotation in patients with cervical dystonia (Asahi et al., 2018). However, all of those studies were based on unilateral HR, and did not investigate the potential mechanism about HR. In the present study, the SEMG test found that the effects of bilateral HR on the myoelectrical activities (aEMG) of SPL was much higher than unilateral HR (Table 2), and further multiple linear regression analysis also showed the HR pattern (no HR = 0, unilateral HR = 1, bilateral HR = 2) was an independent affecting factor for the rotators’ myoelectrical activities (Table 4), which means the bigger the HR pattern (bilateral HR = 2 > unilateral HR = 1), the higher the aEMG. SPL is a deep layer rotator, which is also one of the most powerful rotators of the cervical spine. Our results indicate that the bilateral HR can induce a much stronger muscle contraction of SPL (higher aEMG value) than the unilateral HR, suggesting that bilateral HR has greater clinical potential to be used as a new rehabilitation therapy for cervical neuromuscular disorders than unilateral HR.

The present study also found that the subject’s general characteristics were independent affecting factors for the rotators’ myoelectrical activities. The multiple linear regression analysis showed that age and BMI were independent affecting factors for the aEMG of SPL and SCM, and age was an independent affecting factor for the aEMG of UTr. It suggests that subjects with older age can achieve the higher myoelectrical activities of SPL, UTr, and SCM, and subjects with smaller BMI can achieve the higher myoelectrical activities of SPL and SCM. Similar to our results, many studies have proved that aging factors can affect spinal muscular activity and neuromuscular function (Aubertin-Leheudre et al., 2020). Many cervical disorders are related to aging, for example, NP has a lifetime prevalence rate of 48.5% and is very common in elder people (Cohen and Hooten, 2017), and Shuang et al. (Ao et al., 2019) have pointed out that the subject’s age was the independent risk factor of cervical kyphosis. Hence, the clinical application of HR has a particular significance for elder patients. High BMI is associated with muscular degeneration, for example, the musculature fatty infiltration is a classic indicator of muscular degeneration (Koji et al., 2019), which leads to muscular dysfunction and disorders. Hence, subjects with lower BMI are prone to have more functional cervical rotators. Besides, the present study also calculated the BMI cut-off values. The cut-off value is also called as the “critical value”, which is the critical point obtained from the ROC curve of the subject. We found that the cut-off value of BMI for the aEMG of SPL was 24.37 (Figure 5A), and the cut-off value of BMI for the aEMG of SCM was 23.99 (Figure 5B). It indicates that subjects with BMI more than 24.37 and less than 24.37 can obtain a significantly different efficacy on SPL aEMG during the HR, and subjects with BMI<24.37 are prone to have more functional SPL; subjects with BMI more than 23.99 and less than 23.99 can obtain a significantly different efficacy on SCM aEMG during the HR, and subjects with BMI<23.99 are prone to have more functional SCM. In all, our results indicate that patients with lower BMI and older age can achieve the higher cervical rotators’ myoelectrical activities during HR, which may result in better efficacy and outcome.

CCR is a functional parameter representing muscular coordination and balance. Muscular co-contraction refers to the simultaneous activation of agonists and antagonists in static or dynamic state (Chih-hsiu et al., 2014), and it is an important mechanism for spinal stabilization (Le et al., 2017). In fact, the cervical muscular function is of great significance to spinal stability, it even plays a more important role than the osseous-ligamentous system (Panjabi et al., 1998; Cheng et al., 2011). The rotating CCR is the reference for the coordination of rotators and antagonists in a balanced muscular system. Our result showed the rotating CCR was significantly increased under the unilateral HR comparing to no HR, and it was further increased under the bilateral HR. The increased CCR is considered as a protective mechanism for maintaining the spinal stability during motion (Ford et al., 2008). HR benefits both the rotators’ electrophysiology function and cervical stability. When HR increased the rotators’ myoelectrical activities during active cervical rotation, it can also increase the antagonists’ muscular tension, resulting in a higher CCR and cervical stability. What’s more, the increased CCR also indicated the antagonists’ stretching, which has a great clinical application value for patients with unbalanced musculature and posture, such as cervical dystonia. Higher CCR indicates that the muscular system is in a higher energy consumption model, as the antagonists’ muscular tension is also increased simultaneously with the agonists’ during the rotation, which applies to the systematic muscular exercise. The multiple linear regression analysis showed the HR pattern (no HR = 0, unilateral HR = 1, bilateral HR = 2) was the only independent affecting factor for CCR, which means only the HR pattern can change the CCR, and the bilateral HR can achieve a higher CCR than unilateral HR (bilateral HR = 2 > unilateral HR = 1). Our results indicated that the cervical muscular system (both the agonists and antagonists) contracted much more strongly during the rotation motion under the bilateral HR than the unilateral HR, suggesting that bilateral HR has the greater clinical potential to be used as a non-invasive treatment for cervical neuromuscular disorders related to unbalanced musculature and instability.

There were some limitations of this pilot study. Firstly, the present study only explored the impacts of HR on cervical rotators’ electromyography and neuromuscular function in asymptomatic subjects. Although the pilot study included asymptomatic volunteers as the subjects, it found out the muscular electromyography mechanism of HR, which can be a rationale for the clinical application as a new rehabilitation method for treating cervical neuromuscular disorders. However, in order to identify the effects of HR on the patients, further clinical trials need to be carried out in patients with neuromuscular disorders. Secondly, we only detect the instant impacts of HR on cervical rotators’ EMG, and this pilot study suggests that HR has a greater clinical potential to become a rehabilitation method for treating cervical neuromuscular disorders. However, in order to identify the long-term effects of HR on cervical musculature, further clinical trials with continuous rehabilitation of different HR patterns need to be carried out.

## Conclusion

The present study was a pilot clinical study, which has explored the impacts of HR on cervical rotators’ electromyography and neuromuscular function in asymptomatic subjects. To our knowledge, it was the first study focusing on the electromyography mechanism of HR and its effects on cervical rotators’ function. The present study found that: 1) HR can increase the rotators’ myoelectrical activities of cervical spine, as well as the rotating CCR of cervical muscular system; 2) the effects of bilateral HR pattern on the myoelectrical activity and CCR are greater than unilateral HR pattern; 3) HR pattern, age, and BMI are independent affecting factors for the rotators’ EMG values, and HR pattern is the only independent factor for the rotating CCR. Our study suggests that HR has a greater clinical potential to become a rehabilitation method for treating cervical neuromuscular disorders, and the efficacy of the bilateral HR on cervical neuromuscular disorders maybe stronger than the unilateral HR.

## Data Availability

The raw data supporting the conclusion of this article will be made available by the authors, without undue reservation.
